# Research progress on formation mechanism of pearl

**DOI:** 10.1016/j.heliyon.2024.e35015

**Published:** 2024-07-22

**Authors:** Yingyu Zhang, Shiyu Geng, Guilan Yu, Yijiang Hong, Beijuan Hu

**Affiliations:** a School of Life Science, Nanchang University, Nanchang, Jiangxi Province, China; b Jiangxi Province Key Laboratory of Aquatic Animal Resources and Utilization, Nanchang University, Nanchang, Jiangxi Province, China

**Keywords:** Pearl, Biomineralization, Matrix protein, Metal ions, Organic pigments

## Abstract

Pearls are deeply cherished for their rich color and gorgeous luster, and their quality directly affects their value. Currently, the evaluation of pearl quality is mainly based on four aspects: color, shape, size and smoothness. The quality of pearls is influenced by a variety of factors, categorized into internal factors, such as the structural composition of the nacreous layer and genetic factors of the mussels, and external factors, including the aquaculture environment. Existing research results indicates that genetic factors are the dominant factor controlling the pearl quality. However, the macromolecules such as metal ions, organic pigments and various physical and chemical factors in the aquaculture water environment will also significantly impact pearl quality. Among these, matrix proteins are organic macromolecules found in the nacreous layer that play an important role in pearl quality. They participate in the deposition of calcium carbonate and the construction of the organic framework, affecting the pearls’ size and shape. The color of pearls is influenced by the deposition of metal ions, the transport of organic pigments and the regulation of microstructure.

## 1 Introduction

**Fig. 1:**
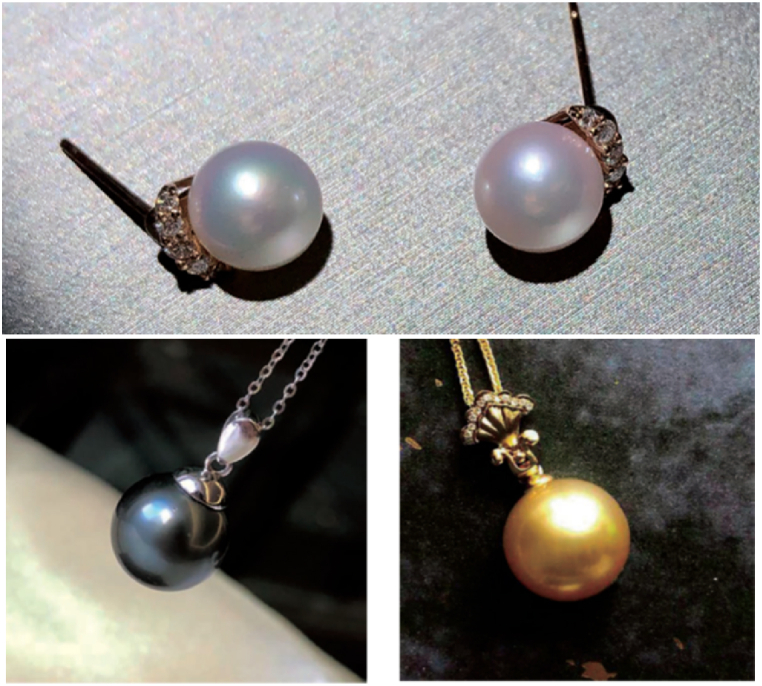
Different colors of pearl (photographs by Liuming) 1. The Akoya pearl 2. The Tahiti black pearl 3. The South sea gold pearl.

Pearls are a kind of organic gemstones valued for its rich color and brilliant luster (Fig. 1). The quality of a pearl is judged mainly from four aspects: color, size, shape and smoothness. The factors that affect the quality of pearl include environmental factors, structural factors and genetic factors. The body color of organisms in nature can be divided into two types: biological color [1] and structural color [2]. Biological color is the reflection of different pigments produced by pigment cells in the biological pigment system. For examples, previous morphological studies on the mantle ultrastructure of various abalone species have reported the presence of pigments within tubular structures located in the mantle region believed to produce the prismatic layer [3,4]. Structural color refers to the refraction, diffraction or interference of light waves caused by the subtle physical structure of the biological body surface [5,6] for example, the rainbow colors seen in the wings of butterflies and mollusk's shells. Pearl color is a comprehensive visual effect presented by its body color, secondary color and halo color [7]. The body color is the background color, which is the color of pearl after selectively absorbing white light [8]. The secondary color is the color floating on the surface of the pearl [9], which is the unique color formed by the reflection and interference of light [10]. Halo color is a kind of floating rainbow color, in which Tahiti black pearl has a strong halo color, which is deeply loved by consumers [11]. The shape and size of the pearl is related to the thickness of the nacreous layer and the deposition mode of calcium carbonate crystal, and the smoothness of the pearl is closely related to the structure of the pearl layer.

The nacreous layer of the pearl is composed of about 95 % calcium carbonate and 5 % organic matrix [12], in which the crystal structure of calcium carbonate is divided into aragonite crystal and calcite crystal, endowing the pearl with unique physical and mechanical properties and luster [13]. The matrix proteins among in the organic matrix construct the organic framework of the nacreous layer and regulate the nucleation and crystallization of calcium carbonate, affecting the morphology, color and luster of the pearl [14]. This paper summarizes the factors that affect the quality of pearls and the research status of related matrix proteins, and aims to explore the specific mechanism of matrix proteins on the regulation of pearl quality.

### 1.1 Mussel and pearl-forming mechanism

There are differences in the pearls produced by different types of pearl oysters. Freshwater pearl mussels include species such as *Hyriopsis cumingii*, *Cristaria plicata*, *Hyriopsis schlegeli*, and *Anodonta woodiana*. These freshwater pearl mussels are abundant in resources, convenient for pearl nucleus insertion by operation, and produce pearls with high yield and quality [15]. Sea-breeding pearl oysters include small pearl oyster like *Pinctada martensii*, and large pearl oysters like *Pinctada maxima* and *Pteria Penguin (Roding)*. These species are found in the South China Sea and produce seawater pearls known as ‘Southern Pearls’ [16], are larger in size than freshwater pearls and better in terms of brightness, secondary color, and halo color [17].

Pearl formation is a process of biological mineralization. Biomineralization refers to the selective use of inorganic elements and regulation of organic matrices (such as carbohydrates, proteins, and lipids) with the participation of the organic matrix. This process leads to the deposition of inorganic elements on organic matter to form the special structure of biominerals [[18], [19], [20]].

### 1.2 Life history of mussel

**Fig. 2:**
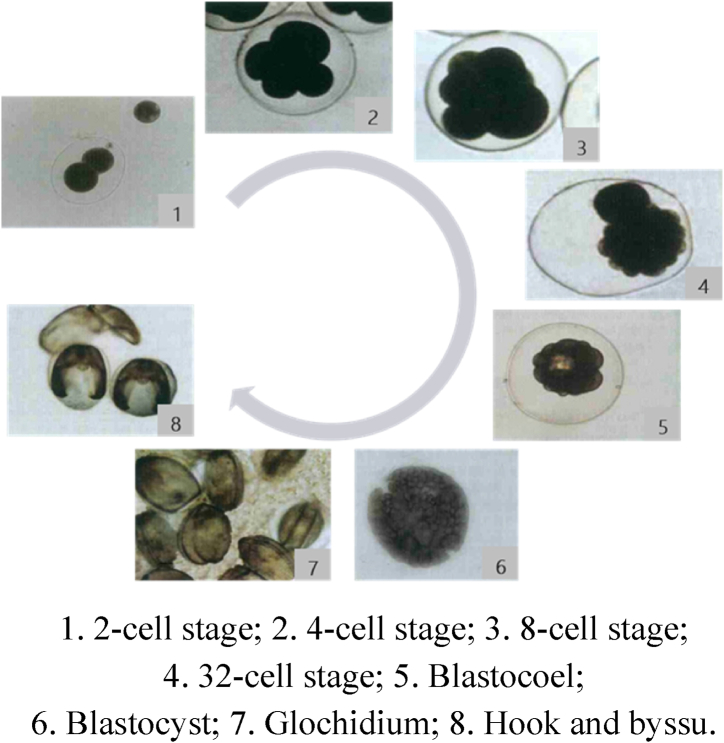
Embryonic development of *Hyriopsis cumingii*. 1. 2-cell stage; 2. 4-cell stage; 3. 8-cell stage; 4. 32-cell stage; 5. Blastocoel; 6. Blastocyst; 7. Glochidium; 8. Hook and byssu.

The development process of freshwater mussel such as *Hyriopsis schlegeli* can be divided into seven stages, they are fertilized egg, cleavage stage, blastocoel, blastocyst, glochidium, juvenile and adult stage (Fig. 2). During reproduction, male mussels excrete mature sperm into the water. The sperm enters the gill cavities of female mussels as they breathe, where they combine with the mature egg cells of female mussels to form fertilized eggs [21,22]. The fertilized eggs develop into glochidium after going through the cleavage stage, blastocoel and blastocyst. The glochidium then leave the female mussels. Eggs develop within the female mussel in a specially modified gill pouch called the nurturing pouch. The glochidium firstly parasitize the gills and fins of fish. After metamorphosis into juvenile mussels, they leave the fish and enter the water as benthic fauna.

The embryonic development of marine mussel is slightly different. The developmental process of *P. fucata* is classified into six stages: fertilized egg, trochophore, veliger (D-shaped stage), pediveliger (umbonal stage), juvenile and adult [23].

### 1.3 The formation of mussels’ shell

The shell structure of mussels is generally composed of three parts, the periostracum, the prismatic layer, and the nacreous layer, all secreted by mantle tissue. Mantle secretion is involved in shell formation. Macromolecular substances such as calcium carbonate, matrix proteins, carbohydrates, and lipids are transported through the hemolymph to the binding sites of biomineralization to control the formation of shells.

There are two ways for mussel to absorb calcium: one is to absorb calcium from food through the digestive system, and the other is to absorb calcium from the environment through organs such as gill and mantle [24]. Ion is difficult to penetrate directly through mantle epidermal cells, so mussel absorbing Ca^2+^ need protein involvement. In the first stage, mantle epithelial cells absorb Ca^2+^ and CO_3_^2−^/HCO_3_^−^ from extrapallial fluid and transport them into vesicles, forming polymer-induced liquid precursor-like amorphous CaCO_3_ granules (PILP-like ACGs) in intracellular vesicles [25]. Subsequently, free ionic calcium will constitute Ca^2+^-protein coacervates by combining with matrix proteins such as Pif80, which are stored stably in the form of CLP. The amorphous CaCO_3_ granules (ACGs) are then transported from the vesicles to the mineralized site by exocytosis. The hyperosmotic extrapallial fluid destroy calcium-induced protein coacervates, causing PILP-like ACGs to become unstable and release Ca^2+^ [26]. The corresponding matrix proteins were involved in regulating the growth of aragonite and controlling the polygonal morphology of nacre aragonite tablets [27].

In the embryonic development stage of freshwater mussels, females provide calcium and other substances to form the shells of the glochidium [28]. Right after the rupture of membranes, the glochidium's shells have many holes of different sizes. The mantle discharge secretions (such as calcium carbonate, carbohydrates, lipids) to fill up the holes, and form intact shells eventually [29].

### 1.4 The composition and formation of nacre

Nacre is composed of 95 % calcium carbonate and 5 % organic matrix. The organic matrix includes biological macromolecules such as proteins, chitins, carbohydrates, and lipids. Proteins play an important role in the regulation of calcium carbonate biomineralization. These proteins are collectively referred to as matrix proteins [30].

The formation process of nacre can be roughly summarized as follows: (1) assembly of the matrix, (2) initial formation of the mineral phase, (3) nucleation of individual aragonite tablets and (4) growth of the aragonite tablets to form mature aragonite crystals. Mantle epithelial cells secret macromolecular substances that regulate the nucleation of free ionic calcium in the cells by combining with carbonate or bicarbonate ions [31].

**Fig. 3:**
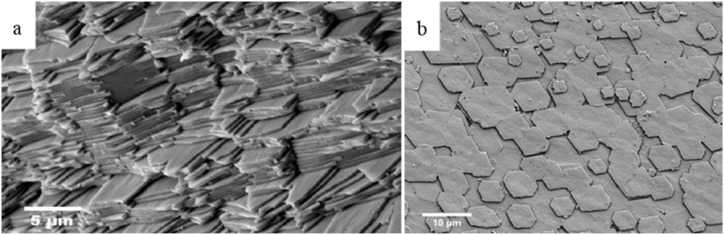
The ‘brick wall’ structure of nacre (a) sectional view and (b)exterior view.

The nacre is an aragonite-protein multilayer. Most of the aragonite crystals are hexagonal, with a thickness of about 0.5 μm and a size of 3–5 μm (Fig. 3) [32]. Aragonite crystals are coated with protein and polysaccharides, this structure formed by calcium carbonate and protein alternately produces interference and diffraction effects on light, which makes the nacre produce a beautiful rainbow color. This brick-wall-style stacking structure gives this layer the special mechanical properties that make the shells resistant to fracture [33].

### 1.5 The formation of cultured pearls

**Fig. 4:**
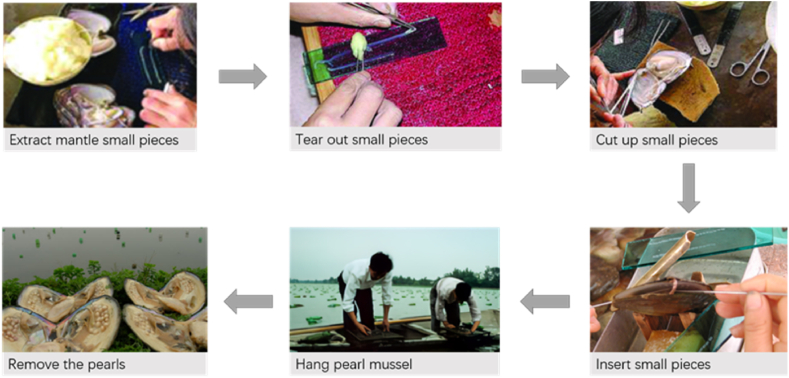
Flow chart of artificial cultivation of non-nucleated pearls.

Artificially cultured pearls mainly include shell pearl, nucleated pearl, and non-nucleated pearl. Freshwater pearls are mostly non-nucleated. When artificially cultivating non-nucleated freshwater pearls, firstly, a mantle piece taken from the mantle epithelial cells of the donor mussels and inserted into the host mussels (as shown in Fig. 4). Within the mussel, mantle pieces develop into pearl sacs during the proliferation and differentiation. The epithelial cells of the pearl sac secrete various ions and biological macromolecules required for biomineralization to form nacre wrapped around pearl nucleus and gradually generate pearls [34]. If the pearl nucleus is inserted into the mussel along with the mantle pieces, it results in a nucleated pearl [35].

## 2 Internal factors affecting the quality of pearls

The quality of pearls is determined by a combination of internal and external factors. Understanding these factors is crucial for optimizing pearl cultivation techniques and enhancing pearl quality. This chapter focuses on the internal factors that influence pearl quality, specifically examining the structural characteristics of the nacreous layer, the age and sex of pearl mussels, and the genetic factors involved.

### 2.1 The structural factors of nacreous layer

#### 2.1.1 The structure of nacre affects the color of pearl

Nacre, the main structure of shells and pearls, is mostly composed of aragonite crystals [36].The surface of the aragonite crystals is coated with organic matter such as proteins and polysaccharides [37]. Yan Jun [38] found that white, purple, and pink pearls all turned milky yellow after mechanical pulverization. Scanning electron microscopy revealed that pink pearls have thinner aragonite tablets near the outer surface and a more compact "brick wall” structure compared to other colored pearls. Ma Hongyan [39] studied the ultrastructural characteristics of the surface of white seawater pearls and concluded that the secondary color of pearls was related to the regularity of growing texture on the surface. A study by Li Qingmei [40] found that the thickness of the aragonite tablets will affect the body color and halo color of pearls.

#### 2.1.2 The structure of nacre affects the luster of pearl

Calcite, aragonite and vaterite are three forms of calcium carbonate crystals. Among the three allotropes, vaterite, a very unstable polymorph of calcium carbonate that rarely appears in nature. Ma Hongyan [41] discovered the existence of vaterite for the first time in the study of matt pearls bred by *Hyriopsis cumingii* in Leidian, Zhejiang Province. It was found that these pearls lack luster, are gray-white, and have a rough texture. At the same time, it was found that matt pearls composed of vaterite can be classified into two types under a microscope: one type has indistinguishable mineral phases due to the mixing of yellowish-brown organic matter, and the other type has larger mineral phases, about 3–15 μm in size, with granular, radial, or irregular shapes.

Through polarizing microscope observations, Cao Lijia [42] found that pearl luster is positively correlated with the layered surface structure. The more homogeneous and compact the structure, the smoother and glossier the pearl. Kong Bei [43] used scanning electron microscopy to study marine cultured pearls, discovering that luster and brightness are determined by structural order, aragonite crystallinity, and keratin membrane thickness. These factors also influence the pearls' transparency, body color, and secondary color.

### 2.2 The age and sex factors of pearl mussel

Through a comparative study on the growth and pearl production performance of male and female *Hyriopsis cumingii* at different ages, Zhao Yongchao [44] found significant differences in the physiological indexes of female and male *Hyriopsis cumingii* from the third year, with corresponding differences in pearl production performance. Overall, male mussels displayed better pearl performance under traditional culture methods, while separating females from males enhanced growth and pearl production in females.

Currently, most pearl production uses both donor and recipient oysters approximately 2–3 years old [45]. Linda Adzigbli [46] examined age's impact on pearl production and immune responses in Pinctada fucata martensii. Younger oysters, around 1.5 years old, showed better performance in both aspects. Chin-Long Ky [47] used grafting and regrafting operations in *Pinctada margaritifera* to assess the impact of donor oyster age on pearl quality traits, recording and comparing six specific traits. The study revealed significant differences between donor oysters of different ages, with younger donor oysters typically yielding higher-quality pearls. Japanese scholars [47] have found that when 2-year-old mussels are selected as donor mussel, most of the pearls are cream-yellow and golden, and the nacreous layer is thick. When 3-year-old mussels are selected as donor mussels, there are more white pearls. When 4-year-old mussels are used as donor mussels, there is a high yield of white pearls but with a poor ability to secrete nacre [48]. Lin Weicai [49] by analyzing the main factors of pearl production performance effect of *Pinctada matensii*, it was found that the pearl production performance value was highest for 2-year-old host mussels and lowest for 3-year-olds. Xu Qiaoqing [50] investigated age-related changes in hemocyte density, phagocytic activity, and immune function in *Hyriopsis cumingii*. Results showed hemocyte density and plasma lysozyme activity increased with age. Overall, the study suggests a link between immunological activity and cultured pearl quality in freshwater mussels.

### 2.3 The genetic factor of pearl mussel

The quality of pearls, including attributes such as color, luster, shape, size, and smoothness, is influenced by genetic factors. Currently, selective breeding of pearl mussels is primarily employed to enhance these qualities. Research on selective breeding programs for pearl mussels has mainly focused on the growth and inner shell color traits that are closely associated with the quality of the resulting pearls, yielding significant advancements. For example, the size and weight of pearls are affected by the host oyster characteristics [51,52]. The color of the pearls is influenced by the color of the donor oyster [53,54] and pearl color and luster are important criteria in defining the quality a pearl. In the 1970s, scientists carried out genetic improvement of *Pinctada martensii*, to reduce the frequency of yellow pearls and increase the yield of silver-white pearls. Through three consecutive generations of mass selection, as a result, the ratio about white mussels increased from 20 % to 80 % [55]. Sun Tianyang [56] found that the inner shell color and luster of the golden strain *Hyriopsis cumingii* could be improved through selective breeding. In 2018, Shanghai Ocean University successfully cultivated a new variety of *Hyriopsis cumingii* named ‘Shenzi No. 1’ characterized by its dark purple nacre. This variety exhibits a high proportion of purple individuals, reaching up to 95.6 %, and can consistently produce purple pearls [57]. According to the above research, genetics play a significant role in determining the quality of pearls.

#### 2.3.1 Current status of transcriptomics research

Transcriptome sequencing technology can sequence all mRNAs transcribed by tissues or cells under specific conditions, without being limited to a single gene [58]. It allows for the study of non-model organisms in the absence of a reference genome, demonstrating significant advantages and application prospects. By using transcriptome sequencing technology to screen for differentially expressed genes (DEGs) that affect cultured pearl quality, a solid foundation is provided for research in areas such as selective breeding, molecular marker development, and the construction of genetic linkage maps [59]. Researchers have utilized transcriptome sequencing technology to sequence the mantle tissues of various mollusks, thereby obtaining numerous genes related to shell formation. These include lectin, shematrins, lysine-rich matrix proteins (KPMPs), mantle genes, Met-rich matrix proteins, secreting calcium-binding proteins, proteases, protease inhibitors, glycine-rich shell matrix proteins, calponin-like proteins, calcineurin-binding proteins, Follistatin-like proteins, and carbonic anhydrase 1, among others [[60], [61], [62], [63]].

The research on genes related to nacre coloration is as follows. Sarah screened three groups of Pinctada margaritifera with completely white, black, and semi-albinistic extreme colors. Through transcriptome analysis, they identified differentially expressed genes and elucidated the genetic processes related to biomineralization and pigment deposition in nacre [64]. Liu [65] identified the key gene srb-39 for carotenoid transport and deposition in the mantle through transcriptome sequencing and RNA interference experiments. William [66] conducted transcriptome sequencing of colored tissues in Calliostoma zizyphinum Linnaeus and found differentially expressed genes related to uroporphyrin production, confirming the relationship between porphyrin pigment deposition and shell color polymorphism. Yue [67] performed transcriptome sequence analysis on the mantles of Meretrix meretrix, revealing the key role of the Notch signaling pathway in shell color regulation. Ding [68] studied different shell colors of Patinopecten yessoensis and found that tyrosine metabolism and betaine biosynthesis are involved in shell color regulation. Feng [69] discovered that the three ABC transporter superfamily members, abca1, abca3, and abcb1, were lowly expressed in white-shelled oysters (Crassostrea gigas). Huang [70] performed transcriptome sequencing on both sides of the mantle of Amusium pleuronectes, finding that 19 genes related to vitellogenesis were significantly highly expressed on the red-shelled side. Teng [71] investigated juvenile Argopecten irradians before and after coloration and found that substances such as porphyrins, metal elements, and melanin might be involved in the shell coloration process. Nie [72] performed transcriptome sequencing on the mantles of white and orange Ruditapes philippinarum, discovering pathways involved in shell color and spot formation, including melanin metabolism, chlorophyll metabolism, calcium signaling pathway, and porphyrin metabolism.

#### 2.3.2 Current status of proteomics research

Pearl quality is mainly influenced by genetic, so the matrix protein (MP) mediated by corresponding gene plays a key role in the regulation of pearl quality. Although the content ofmatrix proteins was less in nacre, they play an extremely important and essential role in the formation of shells and pearls. They can not only affect the growth of crystals but also correcipitation with calcium ions to form biomineral [73,74]. Matrix proteins can construct organic framework, control crystallization velocity and change arrangement of aragonite tablets to affect crystal morphology [75]. Therefore, they may impact on the size, color and luster of pearls.

At present, a large number of matrix proteins have been extracted and identified from mussel. For some species, marine bivalves’ shell matrix proteins have been studied in detail. 115 kinds of shell matrix proteins have been identified in *Pinctada fucata*, 63 kinds of shell matrix proteins have been identified in *Mytilus coruscus* [76], and 113 kinds of shell matrix proteins have been identified in *Mytilus galloprovincialis* [77]. 82 kinds of shell matrix proteins were identified from *Pinctada margaritifera* [78] and 569 species from *Lottia gigantean* [79]. Among freshwater pearl mussels, *Hyriopsis cumingii* has been studied in detail. At present, more than 20 kinds of matrix proteins with complete mRNA sequence were identified and obtained from *Hyriopsis cumingii* by gene cloning, while the studies on related genes and matrix proteins of other freshwater pearl mussels, such as *Cristaria plicata*, *Hyriopsis schlegeli*, and *Anodonta woodiana*, are even more rare. Some of the discovered matrix proteins in mussel are shown in Table 1.

**Table 1:** Some of the discovered matrix proteins in mussel.

Species	Protein name	Structure	Category	Function
*Mytilus coruscus*	GRSP	PDZ (Postsynaptic density/Discs large/Zonula occludens) domain	Prismatic layer matrix protein	Induce morphology change of aragonite CaCO_3_ crystals
		ZM (ZASP-like motif) domain		Inhibit the crystallization rate of CaCO_3_ [80]
	TLP	CH (calponin homology) domain	Prismatic layer matrix protein	Promote the transformation of calcite crystals into aragonite crystals
				Inhibit the crystallization rate of CaCO_3_ [81]
	CLP-2	Two VWA (Von Willebrand factor A) domains	Prismatic layer matrix protein	Induce morphology change of aragonite CaCO_3_ crystals from cubic shape to spherical shape
				Inhibit the crystallization rate of CaCO_3_ [82]
	WLP	PDZ domain	Prismatic layer matrix protein	Induce morphology change of CaCO_3_ crystals
				Inhibit the crystallization rate of CaCO_3_ [83]
*Nautilus macromphalus*	Nautilin-63	Short collagenous-like domain	Nacreous layer matrix protein	Participate in organic framework formation
				Interact with the mineral phase [84]
*Haliotis rufrecens*	Lustrin A	GS (glycine- and serine-rich) domain	Nacreous layer matrix protein	Participate in organic framework formation
				Interact with other matrix proteins [85]
*Haliotis laevigate*	Perlwapin	Three WAP (Whey Acidic Protein) domain	Nacreous layer matrix protein	Adjust CaCO_3_ crystals' shape [86]
*Pinctada maxima*	KRMP-3	Gly/Tyr domain	Prismatic layer matrix protein	Inhibit the crystallization rate of CaCO_3_
				Bind tightly to chitin [87]
*Pinctada fucata*	MSI60	Two alanine-rich domains	Nacreous layer matrix protein	Participate in organic framework formation [88]
	Nacrein	CA (carbonic anhydrase) domain	Nacreous layer matrix protein	Participate in the deposition of CaCO_3_
		N/G-rich domain		The degraded fragment has antibacterial activity [89]
Pteria sterna	N66	CA domain	Nacreous layer matrix protein	Participate in the deposition of CaCO_3_ [90]
		N/G-rich domain		

Species	Protein name	Structure	Category	Function
*Hyriopsis cumingii*	HCCCA3	CA domain	Periostracum, prismatic and nacreous layer matrix protein	Participate in the deposition of CaCO_3_
				Participates in the regulation of coloration
				Relate to pearl weight traits [91]
	HCCUBDC	CUB domain	Nacreous layer matrix protein	Participates in the regulation of coloration [92]
	HCCA2	CA domain	Nacreous layer matrix protein	Participate in the deposition of CaCO_3_
				Participates in the regulation of coloration
				Regulate acid-base balance [93]
	HCTYR	Tyrosinase domain	Nacreous layer matrix protein	Participates in the regulation of coloration [94]
	HCTYP-1	Tyrosinase domain	Nacreous layer matrix protein	Participates in the regulation of coloration [94]
		Chitin binding domain		

#### 2.3.3 Characteristics of matrix proteins

The proportion of one or several amino acid residues (such as aspartate, glycine, and serine) is very high in most matrix proteins [95]. In addition to these amino acids, studies showed that proline (Pro) [96,97], cysteine(Cys) [98], glutamine(Glu) [99], and asparagine(Asn) [100] residues play important roles in biomineralization. The side chain of Asp has a carboxyl that can combine with Ca^2+^ [101]. Amino acid residues such as Asn are relatively high in some matrix proteins and can appear in the form of a repeat sequence. The side chains of Cys and Ser have hydroxyl and sulfhydryl that can participate in the interaction between matrix proteins and other organic macromolecules.

Matrix proteins often have modular structural features. Different modules perform different functions. The most common structural feature is random tandem repeat regions. The filamentous proteins ususally have tandem Ala and Gly sequence, commonly found in the insoluble components of shells. Acidic matrix proteins often have tandem Asp and Glu sequences, beneficial to combine with calcium ion.

Most matrix proteins have their own unique domain. For example, matrix proteins Nacrein and N66 contain carbonic anhydrase domain, which is mainly responsible for catalyzing the conversion of CO_2_/HCO_3_^−^ to CO_3_^2−^, and then reacting with Ca^2+^ to form CaCO_3_ crystals [102]; matrix protein HCTYP-1 contains chitin binding domain, which can assimilate heavy metals. Matrix proteins GRSP and WLP contain PDZ domain, which is a domain involved in protein interaction and biomineralization. Matrix protein TLP contains calponin homology domain, can increase the calcification of fibroblasts, indicating that this protein domain may be related to biomineralization.

#### 2.3.4 The function of matrix proteins

According to whether soluble in EDTA and acetic acid, matrix proteins can be divided into insoluble matrix proteins and soluble matrix proteins: insoluble matrix proteins and chitin participate in the construction of shell hydrophobic organic framework; soluble matrix proteins can fill the organic framework, participate in induction of nucleation, calcium carbonate deposition and crystal growth regulation [103].

##### 2.3.4.1 Participate in CaCO_3_ deposition and control crystal morphology

In the process of regulating shell formation, matrix proteins usually inhibit or promote the crystallization of CaCO_3_ crystals by binding to different crystal plates of CaCO_3_ crystals. By binding matrix protein, CaCO_3_ crystals are induced to have different crystallization rates in different crystal plates, resulting in the formation of CaCO_3_ crystals with different morphologies and crystal forms. The matrix proteins can be divided into three types according to their effect on CaCO_3_ crystallization.

(1) Inhibite the crystallization rate of calcite calcium carbonate. (such as Nacrein, AP7, AP24, N16, Perlwapin and ACLS40);
(2) Promote the crystallization rate of calcite calcium carbonate. (such as PerLucin);
(3) Induce nucleation of aragonite. (such as N66 and N14)

**Fig. 5:**
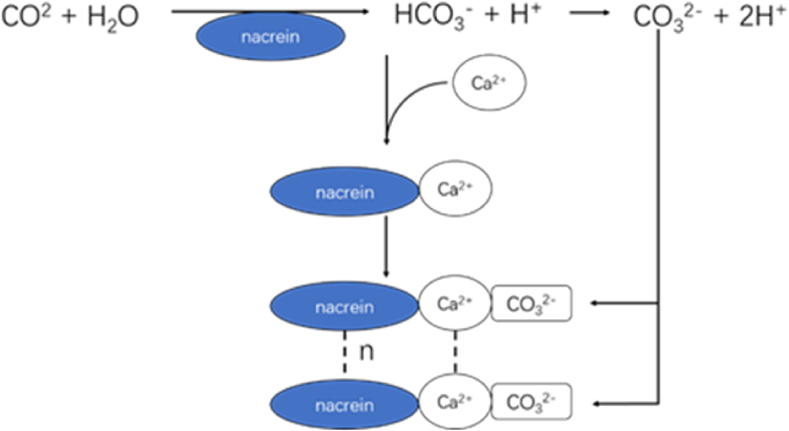
Schematic representation of the nacrein mineralization function.

The matrix protein Nacrein has a carbonic anhydrase domain and a Gly-X-Asn repeat region, in which the carbonic anhydrase domain is mainly responsible for catalyzing the conversion of CO_2_/HCO_3_^−^ to CO_3_^2−^, and then reacting with Ca^2+^ to form CaCO_3_ crystals (Fig. 5), while the structure of the Gly-X-Asn repeat region helps to promote the direct binding of Nacrein to Ca^2+^ and inhibit the crystallization of calcium carbonate [104]. HCCA, HCCA2, HCCA3 and N66 proteins also have carbonic anhydrase domain, which can participate in the crystallization and deposition of calcium carbonate in shells [105].

##### 2.3.4.2 Participate _in_ the construction of organic framework

The nacreous layer is formed by aragonite tablets, and the surface of aragonite is framed by chitin. In the process of mineralization, it is first filled with gelatinous filamentous protein, and then calcium carbonate is mineralized under the action of acidic protein. Among them, Lustrin A [106], Nautilin-63 [107], SILKMAPIN [108], HIC74 [109], and SILKMAXIN [110] are typical filamentous proteins and are important participants in the assembly of organic frames in the nacreous layer.

##### 2.3.4.3 Participate in the regulation of nacreous coloration

Studies have shown that matrix proteins StAR-like [111] and LPCAT1 are involved in regulating carotenoid metabolism to affect nacre coloration. The matrix proteins Creb [112], PKC [113] and HCTYR are involved in regulating melanin synthesis to affect nacre coloration, and HCTYP-1 is involved in regulating the enrichment of metal ions to affect nacre coloration. The matrix proteins HIC6 and HCCUBDC are also speculated to be involved in the regulation of nacre coloration [114].

#### 2.3.5 Current status of integrative analysis of transcriptomics and proteomics

With the development of proteomics, integrating mantle transcriptome or EST library references has made shell proteome and transcriptome correlation analysis a more rapid and efficient research model. Guan [115] used suppression subtractive hybridization (SSH) to screen differentially expressed genes between red-shelled and non-red-shelled pearl oysters, comparing them with a matrix protein database. They identified eight differentially expressed genes homologous to shell matrix proteins, suggesting these genes are involved in shell pigment deposition. Jiang [116] sequenced and assembled the mantle transcriptome using Illumina deep sequencing technology, followed by LC-MS/MS mass spectrometry combined with the transcriptome database to screen for shell matrix proteins in *Perna Viridis*. They identified 54 matrix proteins, including three unique to nacre, laying a foundation for studying the biomineralization mechanisms of different shell structures. Xu [117] combined transcriptomics and proteomics to study individuals of *Pinctada martensii* with different shell colors, discovering differentially expressed genes related to melanin production, retinol metabolism, calcium signaling pathways, and biomineralization. Calmodulin, N66 matrix protein, and nacre protein were upregulated at both mRNA and protein levels, while the glycine-rich protein shematrin-2 was downregulated in multi-omics studies. This provided insights into the relationship between calcium signaling pathways, biomineralization, and nacre pigment deposition. Bao [118] focused on analyzing shell matrix proteins based on sequencing the mantle of *Mytilus coruscus*. The study found that most of homologues were from *Crassostrea, Pinctada, Haliotis*, and *Mytilus*, which showed that *M. coruscus* mantle unigene encoded putative proteins exhibiting sequence similarities with previously characterized SMPs of other Bivalvias, indicating a common originate for these SMPs.

## 3 External factors affecting the quality of pearls

The quality of pearls is not only influenced by internal biological and genetic factors but also significantly impacted by various external environmental conditions. This chapter explores the key external factors that affect pearl quality, including the presence of metal ions, organic pigments, and the methods of nucleus implantation. Additionally, the concentration of calcium, the pH and temperature of the aquaculture water, and seasonal and circadian rhythms play critical roles in pearl development.

### 3.1 Effect of metal ions in aquaculture water environment on pearl

Pearl color is significantly correlated with the type and content of metal ions. Li Lipin [119] showed that the metal elements are slightly different in freshwater cultured pearls with different colors. The content of Fe, Mn, Cr, and other colored elements in white pearls are lower than those in colored pearls. Purple pearls have the highest correlation with Mg and Mn, while the content of Fe in orange pearls is significantly higher than other elements. Ma Hongyan [120] believed that metal trace elements were the main reason for the different body colors of pearls. And the colors of purple and pink freshwater pearls are mainly affected by two metal elements, Fe and Mu. Jiang Yan [121] analyzed pearls with atomic absorption spectroscopy and plasma absorption spectroscopy. They found that there were obvious differences in the contents of Mn, Cu, Zn, Fe, Mg and other trace metal elements in yellow, white and black pearls. Among them, the content of Mn in white pearl was higher, the content of Cu, Zn and Mg was relatively higher in yellow pearl, and the content of Fe in black pearl was relatively high. The content of Hg is the least among the three kinds of pearls. Yang Mingyue [122] used plasma emission spectroscopy to detect trace metal elements in white, orange-red, and purple freshwater pearls. It was found that the contents of metal elements in different colors of pearls were different, and the color of pearls became darker and darker with the increase of Zn, Mg, Ti and V. He Xuemei [123] found that Mg and Zn are related to the color of white pearl, Mg and Fe are related to the color of pink pearl, Fe and Zn are related to the color of purple pearl, and Cu and Zn are related to the color of yellow pearl.

According to the above research, there is a relationship between pearl color and metal element content, but the content of a single metal element does not play a decisive role in pearl color. We find that there are differences in the current research on the relationship between metal elements and pearl color, and it is impossible to determine the corresponding relationship between one or more metal elements and a specific color.

### 3.2 Effect of organic pigments in aquaculture water environment on pearl

Previous studies suggest that organic pigments, such as porphyrins and carotenoids, have a strong relationship with pearl color changes.

Porphyrin is a pigment that is widely found in living organisms. Most porphyrins in nature exist as metal ion complexes, such as chlorophyll and heme. Using fluorescence spectroscopy, Iwahashi [124] found porphyrins in *Pinctada margaritifera*. Wang Jingtao [125] used chemical methods to extract solid colored substances from pearls for the first time, proving that metalloporphyrins were the main substances responsible for coloration using fluorescence spectra and plasma atomic emission spectra. Zhang Yuntao [126] considered that porphyrin and metalloporphyrin are the main reasons for the different colors of pearls.

Carotenoids, the general term for natural polyene pigments, are widely found in various organisms. Studies have shown that mussel contain significant amounts of carotenoids. However, mussel cannot synthesize carotenoids; they must ingest carotenoids from food and store them in their bodies [127]. Urmos [128] found carotenoids in pearls for the first time and speculated that pearl color might be related to carotenoids. Zhang Gangsheng [129] also detected the Raman peaks of carotenoids in the nacres of *Hyriopsis cumingii* and *Pinctada martensii* and speculated that the color of the nacre was related to the carotenoid content. Qin Zuolu [130] used laser Raman spectroscopy to determine the number of conjugated carbon-carbon double bonds in the polyacetylenes of pink and purple high-quality pearls is 10 and 16, respectively. Han Jiwei [131] found that adding 0.000625 % β-carotene to the aquaculture water environment can improve the glossiness of pearls produced by *Hyriopsis cumingii* and promote the regular and orderly crystallization of CaCO_3_ aragonite. Wen Haibo [132] by comparing the content of carotenoids in different tissues of *Hyriopsis cumingii*, it was found that the content of carotenoids was the highest in the liver. In the same individual, the carotenoid content in the mantle with purple inner shell color was significantly higher than that in the mantle in the white area, so it can be inferred that the carotenoid content in the mantle tissue is related to the color of the pearl layer.

By comparing studies on the coloration effects of metal ions and porphyrins, we found that the coloration rules of metalloporphyrins are similar to those of metal elements. The role of porphyrins in nacre is to covalently bind with metal ions. By using Raman spectroscopy to study carotenoids in nacres, we found that pearl color is closely related to carotenoid content. Since carotenoids in mussel come mainly from ingesting algae, we can explore the artificial supplementation of carotenoids to change the color and luster quality of pearls.

### 3.3 Effects of different nucleus implantation methods on pearls

In the study of pearl mussel, mantle pieces from different donor mussels significantly affect the shape and color of pearls. Wada [133] studied the color and weight of pearls by transplanting mantle pieces from pearl mussels with white inner shell. In two experiments, the frequency of yellow pearls produced by transplanting mantle tissue from white pearl mussel was significantly lower than that from brown pearl mussel. Zhang Genfang [134] discovered that the color of non-nucleated pearls in *Hyriopsis cumingii* is determined by the inner shell nacre color of the donor mussels which providing the mantle pieces. Therefore, by selectively breeding new strains with pure purple and pure white nacre, it is possible to directionally cultivate pure purple and pure white pearls. McGinty [135] studied the four combinations of *Pinctada margaritifera* and *Pinctada maxima* as donor oyster and host mussel respectively, it was found that the pearl weight and pearl layer thickness produced by the combination of *Pinctada maxima* as donor oyster and *Pinctada margaritifera* as host mussel were better than those of other combinations. In summary, we found that pearl mussels may have different rejection reactions to different kinds of mantle pieces, which may reduce the quality of pearls produced by their combination, or the interaction between different kinds of donor mussels and host mussels may also combine the advantages of both sides.

Pearl quality is closely related to the site of nucleus insertion. Besides insertion into the mantle, the visceral mass of freshwater pearl mussels also provides a suitable physiological environment for pearl culture. The central space of the man-tle is small, hindering the insertion of large nuclei and the production of large pearls. Conversely, the visceral mass offers ample space for nucleus insertion. Recent research on cultivating large round nucleated pearls in freshwater mussels through visceral mass insertion has shown promising results [136]. Studies have demonstrated that different nuclear positions yield varying outcomes. The survival rate, nucleation rate, and quality nucleation rate are higher at the dorsal and axe foot positions, while other positions result in lower survival and nucleation rates [137].

### 3.4 Effect of calcium concentration in aquaculture water environment on pearl

The calcium concentration in the culture environment significantly affects the growth and development, survival rate, fecundity and pearl deposition velocity [138,139]. Relevant researches show that as the calcium concentration in the culture environment increases, the growth rate of mussels is significantly improved [140,141]. When the calcium concentration in the culture environment is too low, the growth rate, fecundity and shell weight of mussel decreased [142]. Calcium ion is absorbed as the main component of nacrum in the process of shell and pearl formation, but when there is too much Ca^2+^ in the cell, it will have a toxic effect on the maintenance of physiological metabolism in the cell, thus affecting its normal function [143].

Tang Min [144] found that the appropriate of calcium concentration in the water environment can promote the absorption, storage and secretion of calcium. On the contrary, if the calcium concentration in the water environment is too high or too low, it will inhibit the calcium metabolism in the mantle, thus affecting the growth of pearl mussel shells and the formation of pearls. Li Wenjuan [145] studied the effect of different calcium concentration on the accumulation of Ca^2+^ in the mantle tissue of *Hyriopsis cumingii* by laser confocal microscopy. It was found that the calcium concentration in water at 1.25–3.00 mM contributed to the storage of Ca^2+^ in the mussel.

### 3.5 Effects of pH and temperature of aquaculture water on pearls

Qiu Andong [146] studied the mechanism of the effects of five kinds of pH water environment (pH = 5, 6, 7, 8, 9) on nacrum secretion in *Hyriopsis cumingi*i mantle by using a variety of histochemical methods and transmission electron microscopy. The results show that in a neutral water environment, mussels can actively absorb calcium and synthesize and secrete nacre and pearl organic matrix precursors. The higher the degree of acidity or alkalinity of the water environment, the greater the effect on the synthesis and secretion of nacre. Han Jiwei [147] studied the pearl formation of *Hyriopsis cumingii* under different pH levels and found that weakly acidic water (pH = 6) was beneficial to the growth and development of *Hyriopsis cumingii*, resulting in the largest pearls.

Geological research by Cartwright [148] found that the width of nacreous layer of *Pinctada martensii* depends on seawater temperature. An increase in temperature leads to the thickening of aragonite tablets, which, in turn, increases the thickness of the nacreous layer.

### 3.6 Effect of the seasonal and circadian rhythms on pearls

Wada [149] showed that while the growth rate of nacreous layer is fast in summer, but the glossiness is not good. In winter, the growth rate of nacreous layer is slow, but the glossiness is good, indicating that the formation of pearls is seasonal.

Yoko Miyazaki [150] studied *Pinctada martensii* in ocean and aquarium and found that the gene expression of three matrix proteins—Nacrein, MSI60, and N16—involved in the formation of the pearl layer varied between day and night. The gene expression of N16 and MSI60 increased at high tide, while the gene expression of Nacrein increased at low tide, indicating that the process of biomineralization is affected by circadian rhythm.

## 4 Summary

A comprehensive exploration of pearl quality encompasses various internal and external factors, each playing a crucial role in shaping the characteristics of pearls. Internally, the intricate process of pearl formation within mussel involves the secretion of nacre by mantle tissue, leading to the gradual accumulation of aragonite crystals. This process is influenced by factors such as mussel's age, sex, and genetic predispositions. Understanding these internal dynamics provides valuable insights into how pearl size, shape, and luster are determined. Externally, the surrounding aquatic environment significantly influences pearl quality. Metal ions present in the aquaculture water affect pearl color, with different combinations yielding various hues. Organic pigments, including porphyrins and carotenoids, contribute to coloration and luster, with their presence linked to the diet of mussel. Moreover, the method of nucleus implantation, as well as the calcium concentration, pH, temperature, and seasonal variations in the aquaculture water, all impact pearl development.

By considering both internal biological processes and external environmental factors, pearl cultivators can optimize techniques to produce pearls of superior quality. This comprehensive understanding underscores the interdisciplinary nature of pearl cultivation, bridging biology, chemistry, and environmental science to achieve desired aesthetic and commercial outcomes.

## Data availability statement

The data of this study are available upon reasonable request.

## CRediT authorship contribution statement

**Yingyu Zhang:** Formal analysis. **Shiyu Geng:** Investigation, Formal analysis. **Guilan Yu:** Supervision. **Yijiang Hong:** Writing – review & editing, Formal analysis. **Beijuan Hu:** Writing – original draft, Project administration, Methodology, Investigation, Formal analysis.

## Declaration of competing interest

The authors declare that they have no known competing financial interests or personal relationships that could have appeared to influence the work reported in this paper.
